# The Radcliffe Infirmary, Oxford

**Published:** 1895-01-05

**Authors:** 


### EDITOR'S LETTER-BOX.

[Our correspondents are reminded that proxility is a great bar to publication, and that brevity of style and conciseness of statement greatly facilitate early insertion.]

### A GENUINE CITY MEETING.

We take the following note from the *Oxford Chronicle* of 22nd ult. We propose to return to the subject next week, and to show how and to what extent changes are imperative. We hope the popular Mayor of Oxford will call a genuine city meeting without further delay, as the response is sure to be liberal, and so strengthen the hands of the committee and governors:—

I see that The Hospital still keeps on prodding the University, city, and county authorities in the matter of the Radcliffe Infirmary. It wants all Oxford to join in the great work of making "the Radcliffe Infirmary become in the future conspicuous for all that is great in medical science—a school and a hospital worthy of so famous a seat of learning." I don't know so much about the "school" part of the business—that is for the University—but I am quite certain that there is a general desire to place the infirmary in as complete a state of perfection as possible, to obtain buildings for it which shall be thoroughly worthy, and to see that the medical and nursing staffs have every sort of modern convenience. You, Mr. Editor, have often pressed for a genuine city meeting in connection with the matter. Why should not one be called early in the New Year? The Mayor has entered upon a term of office in which, so far as one can see, nothing startling is likely to take place. May I suggest to him to make it memorable by giving Oxford a lead upon this question? He could do it if he would, and he would then have the satisfaction of having accomplished a permanent work, a work more lasting in its results than even that of entertaining such a learned body as the British Association.

